# Evaluating digital triage symptom checker with historical triage-related adverse events

**DOI:** 10.1080/02813432.2025.2563517

**Published:** 2025-09-22

**Authors:** Jonathan Ilicki, Sandra Edman, Joacim Stalfors, Carl Johan Molin

**Affiliations:** a Platform24 Healthcare AB, Stockholm, Sweden; b Institute of Clinical Sciences, Sahlgrenska Academy at the University of Gothenburg, Göteborg, Sweden

**Keywords:** Triage, digital triage, primary care triage, urgency, symptom checker, mobile applications, digital health

## Abstract

**Background:**

Online symptom checkers are increasingly used for diagnostic support and triage. However, evidence on their performance and evaluations with real-world data remains limited.

**Objective:**

The aim of this study was to evaluate the performance of a digital symptom checker using clinical vignettes derived from real-world cases that had previously been incorrectly triaged.

**Methods:**

A patient-facing, rule-based digital symptom checker used in Swedish primary care was assessed in this study. Vignettes were constructed from cases reported to the Swedish Health and Social Care Inspectorate due to erroneous telephone triage. We hypothesized that the digital symptom checker could provide appropriate triage for these cases. Seven physicians independently simulated patients by entering symptoms in the symptom checker based on each vignette. Triage outcomes were assessed against the Swedish National Triage Guidelines (RGS), evaluating the accuracy and the safety of the triage recommendation.

**Results:**

A total of 69 unique vignettes yielded 483 individual trials. After excluding 93 trials due to significant deviations from the original vignette description (adding or omitting symptoms), 390 trials were included in the primary analysis. The symptom checker achieved 91% accuracy (95% CI 88–94%) and 94% safety (95% CI 91–96%).

**Conclusions:**

The symptom checker demonstrated high accuracy and safety when triaging a subset of vignettes based on real-world cases that had been previously erroneously triaged. This study also highlights the difficulties of using vignettes when evaluating symptom checkers. To our knowledge, this is the first study to evaluate such systems using vignettes based on actual patient cases with known triage errors.

## Introduction

The use of digital symptom checkers is increasing [1], as is the digitalization of healthcare. The usage of digital symptom checkers includes providing information for patients regarding symptoms or conditions, automatic gathering of medical history for the physician, diagnostic evaluation (to either the patient or the physician), and triage. Symptom checkers for diagnostic evaluation face several challenges. As an output, symptom checkers often present users with several probable diagnoses related to the symptoms they have reported. Triage, on the other hand, is used to guide the patient as to when and where they should seek care, based on their symptoms [2]. In addition, the diagnostic performance varies significantly and is often low [3–5], potentially as diagnostic decision processes can be complex, often including more aspects than simply evaluating symptoms, such as physical examinations or lab tests. Notwithstanding diagnoses, the potential benefit for patients when using symptom checkers in healthcare most likely lies within the area of triage, since the patient needs to know how to proceed when seeking care [2,6].

Evaluations of symptom checkers with real-world patient cases are rare despite the increasing usage [7,8]. Triage tools are often evaluated using self-designed standardized patient vignettes representing common clinical conditions [9]. This method has several limitations, mainly related to poor representation of patient populations and medical conditions, as well as diversity in how patients present symptoms and interact with the system [7–11]. Also, several studies have raised concern regarding the lack of evidence, benchmarking, and regulations as major obstacles for the implementation of symptom checkers in healthcare, together with poor demonstrated performance and general risk aversion, possibly leading to an increased workload for healthcare professionals [6,7,9,12,13]. Several researchers argue that evaluations with historical, real-world patient cases are needed to increase the external validity of studies on symptom checkers, along with overall better triage performance, if they are going to be beneficial for healthcare [6,7,9,10,12].

In Sweden, the first point of contact with the healthcare system is often the 1177 helpline, a telephone service provided by the Swedish Regions [14,15]. The 1177 helpline, which has been gradually implemented by all public health care providers in Sweden since 2003, uses a computer-supported telephone triage protocol developed by 1177, called Rådgivningsstödet (RGS, the Swedish National Triage Guidelines). The RGS triage protocol is clinician facing, completely symptom based, and results in an urgency advice based on the symptoms reported by the patient. RGS does not suggest possible diagnoses. RGS has been extensively used, handling several millions of patient cases every year. The implementation has led to decreased costs and workload for the healthcare system, and the patients’ compliance with the advice given ranges from 55 to 100% depending on the setting and acuity [16,17].

Despite the extensive use and experience of using RGS, adverse events related to telephone triage do happen [18]. In Sweden, the Swedish Health and Social Care Inspectorate (IVO, a governmental agency) supervises healthcare and healthcare staff. According to the Patient Safety Act, healthcare providers are legally obliged to report adverse events that caused, or could have caused, serious injury or disorder to a patient, to IVO. These reports are publicly available to spread knowledge and prevent similar events from occurring.

The symptom checker evaluated in this study is a digital patient-facing online symptom checker, which is used to triage patients in a primary care setting, and is currently being used by a majority of the Swedish Regions as a digital front door to 1177, complementing the 1177 helpline [19]. The symptom checker utilizes a deterministic rule-based system, where the foundation of the medical logic is grounded in the RGS triage protocol. The patient responds to questions about their chief complaint and symptoms using dynamic questionnaires. This means that the patient gets different sequential questions depending on their previous answers. Subsequently, after completing the questionnaire, the system then triages the patient, resulting in an urgency recommendation to patients as to if, when, and how they should seek care.

Given the concerns and recommendations regarding studies on symptom checkers mentioned above, we decided to use patient vignettes based on historical triage-related adverse events reported to IVO in a prospective experimental study. The rationale for using historical adverse triage events was that these could be considered the closest available proxy for real-world data. The aim of this study was to evaluate the performance of the symptom checker by investigating if it could appropriately triage patient vignettes based on historical adverse triage events. We hypothesized that the symptom checker could provide appropriate triage for clinical vignettes based on historical adverse triage events, according to the RGS triage protocol.

## Methods

### Obtaining patient cases

**Figure 1.**
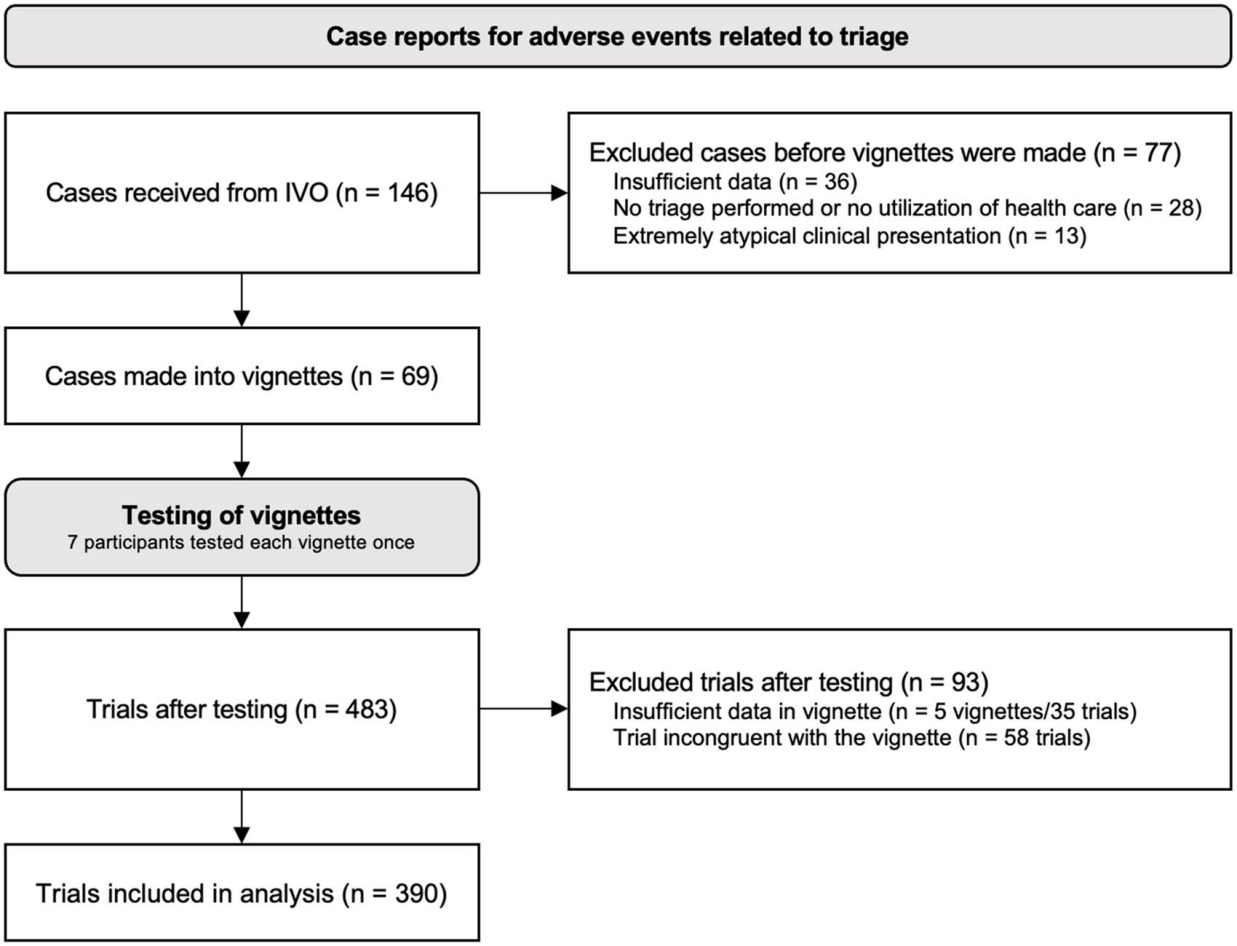
Consort flow diagram of cases, vignettes, and trials. A trial refers to one participant testing one vignette. All participants tested each vignette once. Pre-trial exclusion criteria are defined in Supplementary Table 1. Post-trial exclusion criteria are defined in Table 4.

All adverse events reported to IVO between 1st Jan 2010–18 March 2021, under the category ‘*healthcare advice*’ were retrieved from all regions of Sweden, from which all triage-related events were identified (see Figure 1). For each case, the following data was extracted: patient age and sex, chief complaint, symptom description, previous medical history, current medications, and IVO’s assessment of whether the adverse event resulted in harm or had the potential to cause harm.

### Ethical considerations

No ethical approval was necessary, as reviewed by the Swedish Ethical Review Board, given that no patient-related intervention was made, and the handling of personal data did not fall within the scope of the Swedish Ethics Review Act (Dnr: 2021–03462). The data in the Lex Maria register used in this study is publicly available on request and only contains anonymized patient data.

### Vignette creation

Patient information from the adverse event was used to create patient vignettes. As the symptom descriptions from IVO were often sparse, summarized in medical terminology, and varied in structure, certain terms, such as ‘constitutional symptoms’ or ‘neurological deficits’ were rewritten in lay language to better align with the symptom checker’s patient-facing interface. Cases for which triage was not feasible or where the available information was insufficient were excluded (see Figure 1 and Supplementary Table 1).

Missing data on patient sex and age were generated to produce standardized vignettes. If sex was not reported, random assignment of male or female was applied, except if symptoms clearly implied a certain sex (e.g. patients who were pregnant). If information on age was missing, then a random age between 19 and 90 was applied, except if symptoms or adverse event cases described the patient as a child. If previous medical history or medications were unreported, the patient was assumed to be otherwise healthy and medication-free. No additional clinical details were imputed, and testers were instructed to answer the questions in a way they deemed congruent with the vignette and negate any additional symptoms not mentioned in the vignette.

Each vignette followed the template: *‘[X-year old] [man/woman OR boy/girl] presenting with [chief complaint]. [Description of symptoms]. [Symptom duration when applicable]. [Previous medical history]. [Current medications]’*. The vignettes were written in Swedish to maintain alignment with the original IVO documents. Where possible, the exact phrasing from the Lex Maria register was used. Translated vignettes can be found in Supplementary Table 2.

The RGS triage protocol has five urgency levels (Table 1), ranging from immediate (seeking care right away) to self-care (no need for an assessment right now). As IVO does not state a correct triage recommendation for adverse events, we used RGS to infer the most appropriate urgency based on the chief complaint and presenting symptoms. Three clinicians (two physicians and one registered nurse; SE, JI, CJM) independently assigned urgency levels using the RGS triage protocol as a guideline, and discussed to reach consensus when necessary. To focus on events that would have been possible to prevent, we chose not to include cases with extremely atypical clinical presentations.

**Table 1.** Description of RGS urgency levels.

Urgency	Description
Immediate	Patient should be assessed immediately
Promptly	Patient should be assessed within a few hours
Acute	Patient should be assessed within 24 h
Planned	Patient should be assessed in the next available time slot during office hours
Wait	No need for an assessment right now, self-care advice can often suffice

### Testing procedure

Seven physicians were recruited to test the vignettes. The participants neither had any involvement in the development of the symptom checker nor had access to RGS. Age, sex, and medical seniority were collected for all participants. The participants tested all vignettes using the symptom checker on their own hardware (smartphone or computer). Each participant tested each vignette once. The participants were instructed to seek care using the symptom checker in a test environment, acting as patients presenting with the symptoms described in the vignettes. In this study, one trial refers to one participant testing one vignette. The participants were blinded to all details and origin regarding the original cases, as well as the purpose of the study, but not to the outcome from the trial in the symptom checker. If information on symptom duration was missing in the vignette, they were instructed to assume that the patient had had symptoms for more than 3 h but <1 week, and answer in a congruent manner. Similarly, if information on the level of pain was missing, they were assumed to answer in a way deemed congruent with the vignette. If information on symptom onset was missing, they were instructed to assume that the onset was acute and sudden. If the symptom checker asked for information not described in the vignette, the participants were instructed to answer in a way congruent with the vignette, or negate additional symptoms. Participants were instructed not to discuss the vignettes with anyone.

### Data analysis

Overtriage was defined as a trial resulting in an urgency level *higher* than the level stated in RGS, whereas undertriage was defined as a trial resulting in an urgency level *lower* than stated in RGS. We defined accuracy as the proportion of trials that were triaged to the appropriate urgency level according to RGS. Safety was defined as the proportion of trials that were triaged to the appropriate urgency level according to RGS *or higher*, meaning all trials that were appropriately triaged or overtriaged. 95% confidence intervals were calculated based on the binomial distribution.

All trials resulting in an appropriate urgency level were assessed (i.e. checking stated chief complaint and conditions for the given urgency) to ensure that the triage outcome was reasonable and congruent with the vignette. All trials that resulted in either overtriage or undertriage were examined in detail, checking all reported symptoms and answers given during the trial, to identify trials where participants entered symptoms that were incongruent with the vignette. Trials that met the exclusion criteria (Figure 1) were excluded.

Inter-rater reliability (IRR) was assessed using Gwet’s AC1, as kappa values can result in low values despite high proportions of agreement [20–22], and as Gwet’s AC1 can be used to calculate agreement coefficients for 2 or more raters. Analysis was performed with R version 4.2.2 using the irrCAC package [23,24]. Results were analyzed according to the Altman scale for benchmarking chance-corrected agreement coefficients [25], where an agreement coefficient >0.80 is considered very good, 0.61–0.80 good, 0.41–0.60 moderate, 0.21–0.40 fair, and ≤0.2 poor. The benchmark range membership cumulative probabilities [26] were used for the interpretation of the agreement coefficient.

## Results

### Patient cases and vignette testing

**Figure 2.**
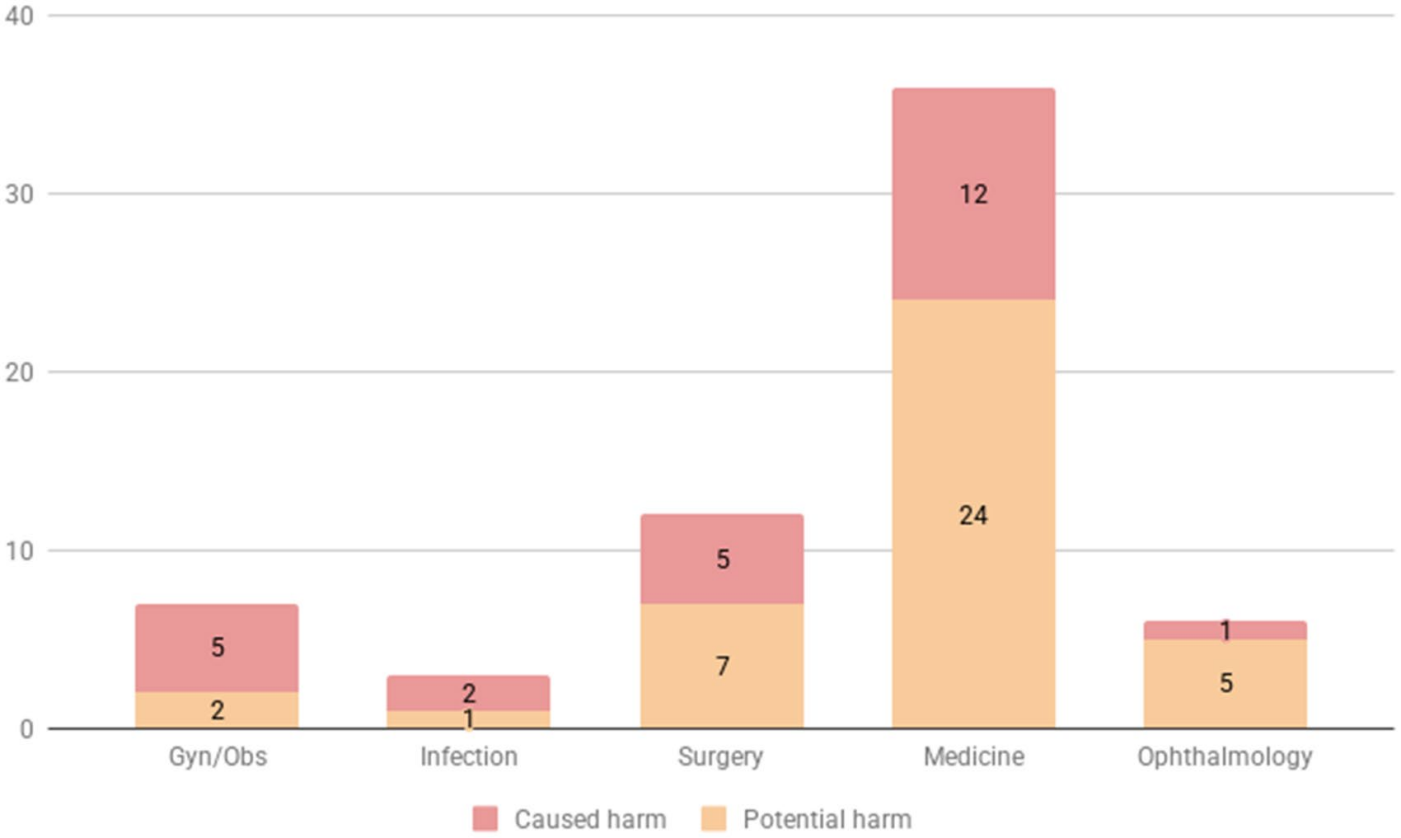
Adverse event cases obtained from IVO, divided by category, and level of harm.

Cases in the category triage/guidance from 2010 to 2021 were obtained from IVO (*n* = 146). A flow chart of cases, vignettes, testing, and exclusion steps is shown in Figure 1. Seventy-seven cases were excluded pre-trial (before vignettes were made). Exclusion categories and examples are presented in Supplementary Table 1. A total of 69 cases remained following pre-trial exclusion, which were made into vignettes. To create an overview of what kinds of medical data was obtained and used for the vignettes, each case was classified by chief complaint into one of five major categories (Figure 2). A majority of cases (75%) related to either medical or surgical conditions. The distribution of urgencies of the obtained cases from IVO is shown in Table 2. Results per tester are described in Table 3.

**Table 2.** Confusion matrix for trial results.

RGS urgency	Cases	Triage results (*n*)					Triage performance	
	*n* (%)	Wait	Planned	Acute	Promptly	Immediate	Accurate	Safe
Wait	0 (0%)	0	0	0	0	0	–	–
Planned	3 (5%)	0	11	7	0	0	61%	100%
Acute	3 (5%)	0	0	14	0	0	100%	100%
Promptly	10 (16%)	0	0	14	48	2	75%	78%
Immediate	46 (74%)	1	5	5	0	283	96%	96%
Total	64						91% [88–94]	94% [91–96]

Urgency levels from trials *vs.* the recommended urgency levels according to RGS. Numbers on triage performance shown as percentage with 95% confidence intervals. Red = undertriage, green = appropriate triage, yellow = overtriage.

**Table 3.** Triage accuracy per tester and tester demographics.

Tester	Trials congruent with original vignette *n* (%)	Triage accuracy (*n*)			Mean AC1
		Undertriage	Appropriate triage	Overtriage	
1	57 (89%)	4	52	1	0.79
2	56 (88%)	3	51	2	0.75
3	57 (89%)	4	52	1	0.81
4	56 (88%)	4	50	2	0.77
5	54 (84%)	4	49	1	0.73
6	55 (86%)	3	51	1	0.79
7	55 (86%)	3	51	1	0.75

For calculation of Gwet’s AC1, all 7 trials of the 64 vignettes were included (*n* = 448). The urgency outcome from each trial (regardless if the outcome was appropriate or not) was used to calculate the AC1 pairwise between each tester and the other 6 other tester. The mean value of these 6 comparisons is presented.

### Post-trial analysis

All seven participants tested each of the 69 vignettes, resulting in a total of 483 trials. After manual review of each trial’s data against the original vignettes, 93 trials were excluded according to the criteria outlined in Table 4. These exclusions consisted of five entire vignettes (35 trials) and 58 individual trials. Most excluded trials were due to incongruent input, i.e. the testers had not correctly reported symptoms according to the vignette. Following these exclusions, 64 patient vignettes remained, corresponding to a total of 390 valid trials. Since individual trials were excluded, not all vignettes resulted in seven trials. The final set of tested vignettes is included in Supplementary Table 2 (in both Swedish and English). In all excluded trials, the triage urgency was appropriate given the symptoms reported during the trial, but as stated previously, the trials were not representative of the original vignettes. A summary of trial results for all vignettes is shown in Table 5.

**Table 4.** Post-trial exclusion categories and explanations.

Exclusion category	Number of vignettes/trials (*n*)	Example of case	Explanation for example
**Lack of information** Vignettes with too vague symptoms to be consistently testable. The definition of this was when the urgency outcome of a trial depended on the user reporting information not defined in the vignette.	5 vignettes/35 trials (5 vignettes × 7 testers)	*26-year-old female presenting with breathing complaints. Has a cough and chest feels heavy. Previously healthy. No medication.*	In order to assess the level of breathing difficulties, the symptom checker asks if the patient has trouble breathing at rest, during walking, during minor physical activity or during normal physical activity. Depending on the answer, the urgency level will vary accordingly, making it impossible to define a single appropriate urgency level for the vignette.
**Erroneous input** Trials where the symptoms stated in the patient vignette were not reported by the participant in the trial, or symptoms that were not stated in the vignette were reported during the trial.	58 individual trials	*42-year-old woman presenting with postoperative complaints. Pain and swelling in arm after cholecystectomy. Has not taken her anticoagulants. Previously healthy. No other medication.*	The appropriate urgency level according to RGS is promptly (assessment within a few hours). One of the testers stated that they had sudden weakness in the arm, which is not stated in the vignette. This resulted in an increase in the urgency level to immediate (assessment right away).

**Table 5.** Summary of trial results, evaluated according to congruency of symptoms reported during the trial.

	Number of trials	Trials resulting in accurate triage	Trials resulting in inaccurate triage	Trials resulting in safe triage	Trials resulting in unsafe triage	Accuracy	Safety
Trials congruent with original vignette (*n*)	390	356	34	365	25	91%	94%
Trials incongruent with original vignette (*n*)	93	93	0	93	0	100%	100%
All trials (*n*)	483	449	34	472	25	93%	95%

All trials include every trial evaluated according to the symptoms reported during the trial, regardless of if this was congruent with the vignette or not. Accuracy = trials resulting in appropriate urgency level according to RGS. Safety = trials resulting in appropriate urgency level according to RGS or higher.

The number of trials congruent with the original vignette is shown per tester in Table 3, and ranged between 84 and 89%. For calculating AC1, the five-tier urgency level outcome from the triage was used. This can be considered a measurement of how well the trials represented the original vignettes, as well as the quality of the vignettes, where a high AC1 value would indicate that the vignettes were easy to follow when inputting data. All trials from the 64 vignettes were included (*n* = 448), regardless of whether the symptoms reported in the trials were congruent with the original vignette. Because incongruent trials that nonetheless resulted in an appropriate urgency level would still be scored as an agreement, these results should be interpreted with caution. Calculated with all trials of the 64 included vignettes, the overall AC1 value for all testers was 0.77. This corresponds to a ‘good’ level of agreement according to the Altman scale, with a cumulative probability of 99.9% for the ‘good’ range (0.6–0.8). When instead analyzing AC1 pairwise between testers, the mean values of each pairwise comparison per tester ranged from 0.73 to 0.81 (Table 3).

### Triage accuracy and safety

**Figure 3.**
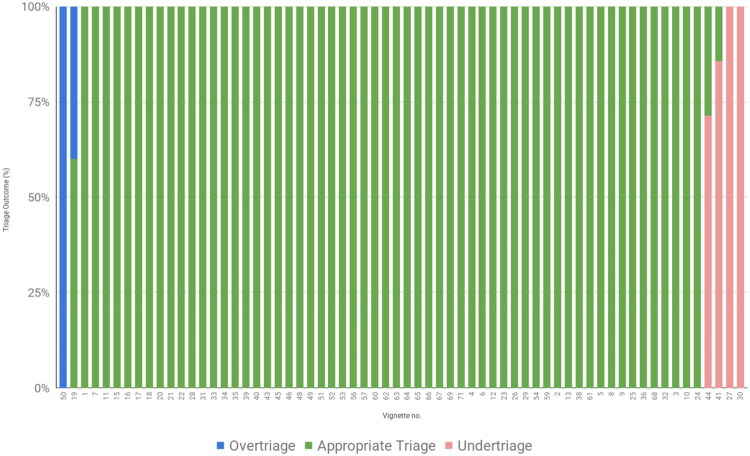
Triage results per vignette. Each bar represents one patient vignette. Colors indicate share of trials per vignette that resulted in undertriage, adequate triage or overtriage.

Out of 390 trials, 356 received an appropriate urgency according to RGS, resulting in an accuracy of 91%. In addition to the appropriately triaged trials, nine trials resulted in an urgency level *higher* than recommended by RGS, resulting in 94% safety. A total of 25 trials received an urgency level which was lower than recommended by RGS (Table 2). As the confusion matrix represents the results of several dependent events, where each vignette was tested by several participants, the share of overtriage, appropriate triage, and undertriage per vignette is shown in Figure 3. The distribution of triage accuracy across testers was very similar, as shown in Table 3.

### Trials with incorrect urgency levels

Two vignettes were identified where the outcome of the trials was too low in 5 and 6 out of 7 trials, respectively. The reason for this was gaps in the medical content, leading to conditioned questions not being asked. Two more vignettes resulted in an urgency that was too low in 7 out of 7 trials. These two cases were identical in terms of medical information (polydipsia or polyuria in children), which, according to RGS should be assessed within a few hours (promptly). In the medical content of the symptom checker, the urgency is deliberately lowered from within a few hours (promptly) to within 24 h (acute) if the patient negates confusion, headache, vomiting, fatigue, weight loss, abdominal pain, and acetone-smelling breath. Further, two vignettes were identified where the outcome of the trials was too high in 2 and 7 out of 7 trials (Figure 3). The reasons for the erroneous triage in these trials were gaps in the medical content, leading to conditioned questions not being asked, or inaccurate escalation of medical severity.

## Discussion

### Accuracy and safety

In this study, the symptom checker had an accuracy of 91% and a safety of 94%. To the best of our knowledge, this is the first study to test digital triage accuracy and safety using vignettes based on real-world patient cases that had previously been triaged inaccurately. By focusing on cases reported to regulatory authorities for incorrect triage, the study addresses a gap in the field, where most prior investigations rely on artificially generated or simulated vignettes [2,5,27–29]. A few exceptions exist—such as a study using NHS Direct call transcripts to develop a subset of vignettes [28], and another using medical records from an Accident and Emergency Department [30], as well as a recent study which suggests that traditional vignettes should be replaced by vignettes generated from actual patient cases [11]. In general, the number of studies on digital triage are few, and most of them evaluate triage for certain conditions or within a specific setting, and/or are not based on real-world patient data [8]. A systematic review comparing the triage accuracy of symptom checkers, LLMs, and laypeople interestingly found that the average triage accuracy was moderate for all three, but the results were more variable within the symptom checker group compared with LLMs and laypeople, which displayed low variability [31]. The review included four studies examining the accuracy of laypeople, ranging from 47 to 62% [11,32–34]. A prospective study comparing triage of a symptom checker with that of patients found that the symptom checker was more accurate (with an accuracy 73%) [32], whereas a study comparing symptom checker triage with nurse triage found 81% agreement, where the nurse triage was considered gold standard [35]. Another study examining a symptom checker in Finnish primary care, with nurse triage as the gold standard, found that the mean of exact match of symptom assessment was 53.7% [36]. As stated above, using clinical vignettes for evaluation above is a more common method for evaluating symptom checker triage. One study [3] found appropriate triage advice in 57.7% when testing 50 vignettes, whereas a review on symptom checkers [6] stated that the triage accuracy for digital online symptom checkers varied between 48.8 and 90.1%. However, the studies included in the review had very different areas of implementation with regards to location, medical specialties, and intended care settings, using both simulated patient vignettes as well as real-world patient data. Also, when comparing the triage accuracy for symptom checkers included in several of the studies in the review, the same symptom checker received very different scores. This highlights the need for open-source, symptom-based clinical vignettes in a standardized format, representing both common and uncommon clinical presentations of various medical disorders. A more recent review [37] on online symptom checkers found that the average triage accuracy varied between 27 and 92%, but once again, the methodologies varied greatly in the included studies. The 45 vignettes originally used by Semigran et al. in 2015 [5] have been reused in several other studies, with varying results [38,39]. Interestingly, a study aiming to replicate the original study by testing the same 45 vignettes with the same symptom checkers found no real improvement in a 5-year follow-up [4]. The same 45 vignettes were also used when comparing triage of symptom checkers with medical laypersons, which found no significant difference between the groups [33]. Interestingly, another study found no difference in triage accuracy when medical laypersons used a search engine (Bing Search API) compared with using a symptom checker (HealthDirect) [40].

Our findings appear more favorable than some previous studies, but methodological and contextual differences limit direct comparability. Vignette studies use different methodologies regarding the design and level of information in the vignettes, as well as the different conditions, case mix, and settings of the vignettes. Furthermore, the definition of reference standard, or ‘correct triage’, differs greatly, where previous research has indicated significantly low IRR when defining the gold standard [11,29,41]. Another difference is that several of the examined symptom checkers in other studies are based on machine learning models instead of deterministic expert systems [28,29,42]. A study that trained a naïve Bayes model on Swedish primary care data to categorize acute *vs.* non-acute conditions reported poor performance, underscoring the challenges of adapting fully ML-based solutions to real-world clinical demands [41]. However, many of these systems have previously been of low maturity, but the recent emergence of publicly available large language models might lead to an increase in their triage accuracy. Recent studies show relatively good triage accuracy, despite that these models are not explicitly trained for medical triage [43–45].

Medical conditions can present with atypical symptoms, and no triage system can have 100% sensitivity for all outcomes without resulting in a large degree of overtriage. Likewise, some degree of undertriage is to be expected in order for the system not to be overly risk averse. We chose to exclude historical cases with an extremely atypical clinical presentation to focus on events that would have been possible to prevent. As the Lex Maria dataset consists of adverse events, it contains a disproportionate number of cases where the initial symptoms may have a spurious connection to a final diagnosis (e.g. presenting with a headache, and later obtaining a diagnosis of cervical tendinitis and acute myocardial infarction). The rationale behind removing these cases was that if the clinical presentation was extremely atypical and not within the scope of RGS, then the event was deemed not possible to prevent. An example of this is provided in Supplementary Table 1. One could argue that these cases most likely have been correctly triaged given their symptoms at the time of the triage, but the final diagnosis was of such a nature that they should have been triaged differently. That these cases exist highlights the diversity and complexity in health care, specifically regarding triage. Although this could be interpreted as excluding cases hard to triage, one should keep in mind that no studies conducted with figured vignettes contain clinical presentations that are not representative of the condition that is being triaged.

It is important to clarify that the RGS decision support is not a single, unified algorithm, but a comprehensive set of over 180 symptom-specific protocols designed for clinician use during telephone triage. These protocols are static, symptom-based, and intended for professional interpretation rather than direct patient use. In developing the symptom checker, the medical logic was indeed grounded in the RGS, but the transition from a static, paper- or screen-based professional tool to an interactive, patient-facing digital application introduces significant complexity. The symptom checker must accommodate layperson input, dynamic questioning based on previous answers, and account for permutations across a wide range of interconnected symptoms and user interpretations. This results in a large number of possible input pathways and recommendations, far beyond the linear pathways of RGS. Due to these factors, it is not reasonable to expect the symptom checker to achieve 100% accuracy when evaluated in regards to RGS. Differences in user input, question interpretation, and the interactivity of the digital format naturally lead to some discrepancies between the outputs of the two systems.

### Implications for clinical practice and development of digital triage systems

Accuracy in symptom checkers is of utmost importance for safeguarding patient health and optimizing healthcare resource allocation. Undertriage can delay necessary interventions for critical conditions, whilst overtriage strains emergency departments and primary care centers, and could potentially have indirect negative effects on patient health due to reduced availability of healthcare resources. However, symptom checkers could be of great use for laypersons, as previous studies have shown that non-medically trained persons tend to overestimate the need for seeking care, while at the same time missing true emergencies [46–48]. High levels of public trust in these tools could mitigate these risks, but only if symptom checkers maintain robust accuracy and reliability. On the other hand, triage systems tend to be quite risk averse, which, instead of decreasing the workload of healthcare personnel causes task shifting from the system and an increased workload. Several studies have discussed this and come to the conclusion that online triage tools should not be recommended, and that a well-defined standard for quality and implementation, as well as regulation, is needed for the systems to be of use [6,7].

Defining a gold standard for the correct triage outcome is not easy. One could argue that the term gold standard shouldn’t be used in the context of triage due to the difficulties in defining a correct triage outcome, and that the term reference standard should perhaps be used instead. A review on emergency triage stated that it’s never possible to have a correct answer for triage of any individual patient, that a ‘correct’ answer probably does not exist, and thus there is no gold standard to use as a benchmark [49]. Nonetheless, a common method for determining the ‘correct’ triage is by the majority vote from a group of expert clinicians [2,41], but this method has significant drawbacks, one of many being that the level of agreement is very low [41,49–51]. In this study, the reference standard was the urgency level recommended by RGS. Although the advice in RGS is also based on advice from clinical expert groups, the system has been in use for almost 20 years, and is constantly revised and improved [52], which makes it a more relevant gold standard than the majority vote system from a few physicians. It should be noted that RGS is designed for use as a decision support for triage used by registered nurses, and not for digital self-triage. Even though RGS have been extensively used, there are still situations where the RGS criteria would benefit from being more detailed, at least when adapted to a digital setting. This caused a few of the trials to be excluded in this study, since the symptom checker in these cases differ from RGS. Still, the RGS triage protocol is used by all public health care providers in Sweden, and thus are suitable to use as a gold standard when defining the appropriate triage in this study.

A study on malpractice claim calls within the 1177 telephone triage found that the main reasons for error were failure to follow the decision support or failure to listen to the caller [18]. While a digital triage system is inherently more binary and will never pick up on the nuances and clinical observations in the way a healthcare practitioner can during a telephone call, both sources of error will never be an issue using a deterministic digital triage system—it will never forget to ask a question, and the same input will always render the same output.

### Limitations

A significant limitation of this study, but nonetheless also a noteworthy finding, was the substantial variability in testers’ ability to adhere to the patient vignettes. The tendency among testers to both omit and add symptoms not present in the original vignette descriptions resulted in the exclusion of a significant number of trials, as they could no longer be considered valid representations of the original vignettes. This observation underscores a key methodological limitation in the use of patient vignettes as a means of evaluating symptom checkers and highlights the need for a standardized framework to guide such assessments. This has been discussed in a study which found that increased standardization of vignettes improved the agreement between testers [53].

Several vignettes and trials were excluded in this study due to e.g. incongruent input and insufficient vignette information. A triage system must be able to handle data with some degree of uncertainty and incompleteness to be of clinical relevance [7]. We aimed to assess this by letting testers fill in the gaps to some extent, as not all symptoms in the vignettes were defined in detail. However, this caused a large number of variations of each case, which in several trials resulted in a combination of symptoms which was no longer representative of the original vignette. The number of excluded incorrect trials due to incorrectly reported symptoms was quite large, which could have been prevented with clearer and more detailed vignettes. As a result, the excluded trials may limit the generalizability of our findings, as they potentially underestimate the real-world variability encountered by patient users. In future studies, creating clearer and more detailed vignettes and providing more structured tester instructions could reduce such exclusions and improve the external validity of the results. Still, from a medical point of view all excluded trials were given an appropriate urgency given the symptoms that were reported in the trials, as stated above.

The results from this study should be interpreted with caution. Safety and accuracy metrics should not be interpolated to the system as a whole, as the study examined a small number of special cases in which the real-world triage have resulted in an incorrect judgement and caused, or had the potential to cause, real patient harm. Furthermore, this study has several limitations related to both external and internal validity. With respect to the external validity, the number of excluded cases before testing was quite large (*n* = 77), mainly due to lack of structured documented data in the reports from IVO for each case. This highlights the need for structured clinical data on historical adverse events for them to be useful for future evaluations. Also, five additional cases were excluded in the post-trial analysis. Although we aimed to test permutations of vignettes by allowing some variation, the clinical information in these vignettes proved to be too sparse, making it impossible to determine the appropriate urgency level based on RGS. Ideally, these cases should have been excluded pre-trial. The number of individual trials removed due to incongruent input was also quite high. A possible explanation for this is testing fatigue, and perhaps more structured vignette data could have made inputting data easier. The number of incongruent vignettes did not differ considerably between testers, as shown in Table 3. Nonetheless, this is reflected in the mean AC1 value of 0.77, which could be considered a measurement of how well the testers succeeded in correctly inputting the vignettes.

Although the vignettes were based on real-world data, the collection of vignettes represents a subset of cases. In this subset, the number of immediate cases was quite high (72%). To better evaluate the performance, the distribution of urgencies in the included cases should be somewhat more even, and ideally also include cases which previously had been correctly triaged. This was, however, not possible in this study due to the nature of the Lex Maria register. As the evaluated subset only contains incorrectly triaged cases, it’s not possible to evaluate the sensitivity or the specificity of the symptom checker. Ideally, a test set including real-world patient cases consisting of both correctly and incorrectly triaged patients would be of great use, since incorrectly triaged cases tend to be skewed towards higher urgencies.

The removal of cases with extremely atypical clinical presentation, which would not have been possible to prevent if using the RGS triage protocol, could also be considered a limitation. While no triage system will, nor is intended to, prevent or identify all possible dangerous conditions due to the inherently chaotic nature of medicine, the removal of cases will increase the accuracy and safety of the results. While these cases might not be possible to prevent, these patients still present in the clinical reality. However, as stated previously, we chose to focus on cases that would have been possible to prevent according to RGS.

Since the cases reported to IVO represent (potentially) serious events, a risk avert system will perform better than when assessing cases that are not as urgent. Moreover, the gold standard was defined using a specific triage urgency level according to RGS rather than a range. Considering the variation of user input, one could argue that a gold standard range might be more useful to assess the results. However, most other digital triage systems have been evaluated using a three-tier urgency scale (emergent care, non-emergent care, and self-care), which is a simplification of the clinical reality, and will also inevitably increase the measured accuracy of the evaluated symptom checkers.

As mentioned in the introduction, the use of clinical vignettes is always limiting. First, the vignettes may not be representative of the conditions and/or population that is being examined. Second, the phrasing might be adjusted to suit the app being examined, and is most probably not similar to the way a patient would present their symptoms. However, we tried to mitigate these factors by using the reports by IVO as a firm template, adjusting the clinical summary as little as possible.

This study also has several limitations in internal validity. Notably, we used physicians, rather than patients, to enter symptoms into the symptom checker. We chose this approach because a substantial portion of previous work on symptom checkers has also relied on physicians as inputters [4,5,28,42], which allows for greater consistency in vignette interpretation and facilitates comparison with existing literature. In contrast, some studies have used symptom entry by a single individual, often one of the authors [38,39]. However, the tested symptom checker is designed to be patient facing, and input from physicians is likely to be different from input by patients, both in a simulation environment and in real-world entry. Physicians are more likely to correctly extrapolate information from the vignette, and are possibly also better at understanding the terminology used. This may lead to physicians envisioning a representative patient case when inputting symptoms, which could lead to less variation and fewer permutations in trials. There is also some evidence that laypeople are less suited for reporting symptoms they never have experienced, which might support the use of physicians rather than laypeople when testing vignettes ranging over several different diagnoses and symptoms [54]. However, given the high degree of variation and the number of errors with regards to trials not being congruent with the vignettes, physicians might not be superior when inputting information. The problem might be inherent to the vignette methodology itself, regardless of whether laypersons or medical staff are inputting symptoms. Nonetheless, using non-medically trained people is most likely the best proxy for real-world symptom checker users [51]. Although consistency with prior research justified the use of physicians for this initial study, we recognize this as a limitation affecting both the internal and external validity of our results.

The lack of structured and comprehensive data on patients’ exact descriptions of their symptoms at the time of their presentation made it difficult to know how well we replicated the historical cases. Also, since the IVO reports were created by healthcare professionals, the vignettes do not accurately reflect the language used by a typical patient.

## Conclusions

This is the first study to assess digital triage using historical patient cases related to previous inaccurate triage causing adverse events. The main finding was that the symptom checker provided appropriate and safe triage corresponding to the RGS algorithm for a subset of reported cases that previously had been inaccurately triaged. The accuracy and safety were 91 and 94%, respectively. Perhaps the most interesting observation was the considerable difficulty testers had in adhering to the original vignettes, which resulted in substantial variability in the number of usable trials. This highlights a critical methodological challenge in using patient vignettes to evaluate symptom checkers: namely, the risk of inconsistent interpretation and representation. These findings underscore the need for more robust and standardized approaches—ideally incorporating real-world data with verified clinical accuracy when assessing the performance and safety of digital triage tools.

## Supplementary Material

Supplementary material.docx

## Data Availability

The vignettes used in the study (in Swedish and translated to English), as well as the appropriate urgency according to the RGS guidelines, are available in Supplementary Appendix 1.
