# Supplementary material for: Evaluating digital triage symptom checker with historical triage-related adverse events
Source: Scand J Prim Health Care. 2025 Sep 22;44(1):1–14. doi: 10.1080/02813432.2025.2563517 (PMC12918410; doi:10.1080/02813432.2025.2563517)
Supplement: Supplementary material.docx [file IPRI_A_2563517_SM7615.docx]

### Supplementary material

| **Supplementary table 1***.* Examples of excluded patient cases prior to testing. | | |
| --- | --- | --- |
| **Exclusion category** | **Number of excluded cases** | **Examples of patient cases received from IVO** |
| 1. Insufficient patient background data for creating a case, or insufficient patient symptom data for determining what urgency level was appropriate according to RGS. | 36 | 1. *Previous healthy woman with a chief complaint of reflux not responding to OTC. Unclear if she had chest or abdominal pain, or any other symptoms. Received a booked time at her GP three hours later. Was later found to have had a myocardial infarction.* 2. *Man with unknown age that seeked telephone advice for unknown symptoms. Nurse did not follow triage guidelines and the patient passed away.* |
| 2. Not possible to create vignette as adverse event doesn’t contain information on triage process. | 28 | *A child that visited the ED repeatedly for fever and vomiting. Was assessed to have a viral infection, and subsequently diagnosed with appendicitis. No documentation of symptoms that were presented for triage.* |
| 3. Not possible to prevent the adverse event due to extremely atypical clinical presentation. | 13 | *A man called his GP clinic due to a headache that had gotten worse and disturbed his sleep during the past week. Previous medical history of osteoporosis, hypertension and a previous intracerebral hemorrhage. Received advice to call the following day to book a consultation. Final diagnosis of Myocardial Infarction with Non-Obstructive Coronary Arteries (MINOCA) as well as cervical tendonitis.* |

| **Supplementary table 2.** Patient vignettes used in the study and translated English vignettes. | | |  |
| --- | --- | --- | --- |
| **Nr** | **Swedish** | **English** | **Urgency** |
| 1 | 33-årig man som söker för buksmärta. Akut insättande kraftiga smärtor i övre delen av buken. Högt sittande buksmärtor. Kräkning, kallsvettig, kan ej sova. Haft besvären i ett par timmar. Tidigare sjukdomar: Frisk. Mediciner: Inga. | 33-year-old man presenting with abdominal pain. Sudden onset of severe pain in the upper part of the abdomen. Vomiting, cold sweats, can't sleep. Has had symptoms for a couple of hours. Previous illnesses: Healthy. Medication: None. | Immediate |
| 2 | 59-årig man som söker för olikstora pupiller. Hängande ögonlock, lite huvudvärk. Haft besvären i ett par dagar. Tidigare sjukdomar: Frisk. Mediciner: Inga. | 59-year-old man presenting with differently sized pupils. Droopy eyelids, slight headache. Has had symptoms for a couple of days. Previous illnesses: Healthy. Medication: None. | Immediate |
| 3 | 43-årig kvinna som söker för synpåverkan. Bubbla i vänster öga, i synfältet. Symptom förvärras. Tidigare sjukdomar: Inga. Mediciner: Inga. | 43-year-old woman presenting with visual impairment. Floater in left visual field. Worsening symptoms. Previous illnesses: None. Medication: None. | Acute |
| 4 | 67-årig man som söker för diarré. Har blodiga och slemmiga diarrer. Tidigare sjukdomar: Inga. Mediciner: Inga. | 67-year-old man presenting with diarrhea. Bloody and slimy diarrhea. Previous illnesses: None. Medication: None. | Acute |
| 5 | 11-årig pojke som söker för ökad törst. Stora urinmängder. Även kräkning, buksmärta. Haft besvären i en vecka. Tidigare sjukdomar: Inga. Mediciner: Inga. | 11-year-old boy presenting with increased thirst. Increased urination. Also vomiting, abdominal pain. Has had symptoms for a week. Previous illnesses: None. Medication: None. | Promptly |
| 6 | 45-årig man som söker för ont i matstrupen. Ont i matstrupen, domningar i armar och händer. Aldrig haft tidigare. Bättre när han andas djupt. Haft besvären i 30 minuter. Tidigare sjukdomar: Frisk. Mediciner: Oklart. | 45-year-old man presenting with pain in lower throat. Also has numbness in arms and hands. Never experienced similar symptoms before. Symptoms are relieved when he takes deep breaths. Has had symptoms for 30 minutes. Previous illnesses: Healthy. Medication: Not known. | Immediate |
| 7 | 86-årig man som söker för bröstsmärta. Diffusa bröstsmärtor med utstrålning mot halsen. Tidigare sjukdomar: Inga. Mediciner: Inga. | 86-year-old man presenting with chest pain. Diffuse chest pain with radiation to the neck. Previous illnesses: None. Medication: None. | Immediate |
| 8 | 81-årig kvinna som söker för bröstsmärta. Värk i vänster arm 3 dagar tidigare samt ont till vänster i bröstkorgen 2 dagar tidigare. Vid samtalet ingen värk eller allmänpåverkan. Haft besvären i ett par dagar. Tidigare sjukdomar: Frisk. Mediciner: Inga. | 81-year-old woman presenting with chest pain. Pain in the left arm 3 days earlier, and left-sided chest pain 2 days earlier. No symptoms during the telephone consultation. Has had symptoms for a couple of days. Previous illnesses: Healthy. Medication: None. | Promptly |
| 9 | 80-årig kvinna som söker för smärta i fot. Har smärta i vänster fot, VAS 9. Kan ej stödja på benet. Blå, kall, svullen marmorerad upp till knä. Haft besvären i ett par dagar. Tidigare sjukdomar: Multisjuk. Mediciner: Inga. | 80-year-old woman presenting with foot pain. Has pain in the left foot, pain rated 9/10. Cannot weightbear. Blue, cold, swollen, mottled skin up to the knee. Has had symptoms for a couple of days. Previous illnesses: Multiple comorbidities. Medication: None. | Immediate |
| 10 | 43-årig kvinna som söker för blodig avföring. Färsk, ibland riklig, blödning från tarmen. Lös avföring. Haft besvären sedan lång tid tillbaka. Tidigare sjukdomar: Polymyalgia reumatica. Mediciner: Prednisolon, methotrexate. | 43-year-old woman presenting with bloody stool. Bright red, sometimes heavy, bleeding from her rectum. Has had loose stools. Has had symptoms for a long time. Previous illnesses: Polymyalgia rheumatica. Medication: Prednisolone, methotrexate. | Acute |
| 11 | 41-årig kvinna som söker för svaghet i arm. Haft besvären i cirka 12 timmar. Tidigare sjukdomar: Hypertoni, osteoporos, hjärnblödning. Mediciner: Inga. | 41-year-old woman presenting with weakness in one arm weakness. Has had symptoms for about 12 hours. Previous illnesses: Hypertension, osteoporosis, cerebral hemorrhage. Medication: None. | Immediate |
| 12 | 86-årig kvinna som söker för svart avföring. Behandlas med blodförtunnande läkemedel och har haft kolsvart avföring 5-6 gånger. Haft besvären i cirka ett dygn. Tidigare sjukdomar: TIA. Mediciner: Clopidogrel. | 86-year-old woman presenting with black stool. Treated with blood thinners and has had black stools 5-6 times. Has had symptoms for about a day. Previous illnesses: TIA. Medication: Clopidogrel. | Promptly |
| 13 | 2-årig flicka som söker för utslag. Hög feber och hudutslag som "massa små hudblödningar" som sprider sig från fötterna och upp mot underbenen. Besvären uppkommit hastigt. Tidigare sjukdomar: Frisk. Mediciner: Inga. | 2-year-old girl presenting with a rash. High fever and a skin rash resembling "lots of small bleeds in her skin", which is spreading from her feet to her lower legs. Acute onset. Previous illnesses: Healthy. Medication: None. | Immediate |
| 14 | 9-årig flicka som söker för viktnedgång. Även trötthet, ökade urinmängder och ökat vätskeintag. Kissar nu även nattetid. Farmor har diabetes. Haft besvären i ett par månader. Tidigare sjukdomar: Inga. Mediciner: Inga. | 9-year-old girl presenting with weight loss. Also fatigue, increased urine output and increased fluid intake. Has also started peeing at night. Grandmother has diabetes. Has had symptoms for a couple of months. Previous illnesses: None. Medication: None. | Promptly |
| 15 | 7-årig pojke som söker för smärta i pung. Akut/plötslig smärta i buken och ena testikeln. Vid samtal med DSK har smärtan i pungen släppt men fortfarande buksmärta. Haft besvären i ett par timmar. Tidigare sjukdomar: Frisk. Mediciner: Inga. | 7-year-old boy presenting with scrotal pain. Acute/sudden pain in the abdomen and one testicle. When talking to primary care nurse, the pain in the scrotum has subsided but still has abdominal pain. Has had symptoms for a couple of hours. Previous illnesses: Healthy. Medication: None. | Immediate |
| 16 | 55-årig kvinna som söker för blod i urinen. Synligt blod i urinen vid tre tillfällen igår. Ingen feber, smärta eller trängningar. Mår bra i övrigt. Haft besvären i ett dygn. Tidigare sjukdomar: Frisk. Mediciner: Indivina, Kalcipos-D forte. | 55-year-old woman presenting with blood in the urine. Visible blood in the urine on three occasions yesterday. No fever, pain or urinary urgency. Feels well besides that. Has had symptoms for a day. Previous illnesses: Healthy. Medication: Hormone replacement therapy, calcium and vitamin D treatment for osteoporisis. | Planned |
| 17 | 56-årig man som söker för bröstsmärta. Symtom från bröstkorg och armar. Haft besvären i 10 minuter. Tidigare sjukdomar: Inga. Mediciner: Inga. | 56-year-old man presenting with chest pain. Has symptoms in chest and arms. Has had symptoms for 10 minutes. Previous illnesses: None. Medication: None. | Immediate |
| 18 | 42-årig kvinna som söker för besvär efter operation. Smärta och svullnad i arm efter gallop. Inte tagit sin blodförtunnande medicin efter op. Tidigare sjukdomar: Inga. Mediciner: Inga. | 42-year-old woman presenting with discomfort after surgery. Pain and swelling in arm after gallbladder removal. Didn't take prescribed anticoagulants after the surgery. Previous illnesses: None. Medication: None. | Promptly |
| 19 | 9-årig pojke som söker för smärta i höger testikel. Tidigare sjukdomar: Inga. Mediciner: Oklart. | 9-year-old boy presenting with right testicular pain. Previous illnesses: None. Medication: Unclear. | Immediate |
| 20 | 46-årig man som söker för kraftiga bröst/buksmärtor. Tidigare sjukdomar: Inga. Mediciner: Inga. | 46-year-old man presenting with severe chest/abdominal pain. Previous illnesses: None. Medication: None. | Immediate |
| 21 | 7-årig flicka som söker för misstänkta vattkoppor. Blåsor på kroppen, feber. Haft besvären i uppskattningsvis ett par dagar. Tidigare sjukdomar: Immunbristsjukdom. Mediciner: Inga. | 7-year-old girl being presenting with suspected chickenpox. Has blisters on the body and a fever. Has had symptoms for approximately a couple of days. Previous diseases: Immunodeficiency disorder. Medication: None. | Immediate |
| 22 | 33-årig kvinna som söker för buksmärta och sammandragningar i vecka 36+4. Gravid efter IVF. Hård och spänd buk. Tidigare sjukdomar: Immuniserad, haft DVT, är APC-resistent. Mediciner: Fragmin. | 33-year-old woman presenting with abdominal pain and contractions at 36 weeks pregnant. Pregnant after IVF. Hard and tense abdomen. Previous illnesses: Rh immunized. Previously had a DVT. APC resistence. Medication: Low molecular weight heparin. | Immediate |
| 23 | 43-årig man som söker för ökade astmabesvär och hosta. Ingen feber, men förkyld. Haft besvären i några veckor. Tidigare sjukdomar: Astma, hypertoni. Mediciner: Inga. | 43-year-old man presenting with increased asthma symptoms and cough. No fever, but has a cold. Has had symptoms for a few weeks. Previous illnesses: Asthma, hypertension. Medication: None. | Promptly |
| 24 | 28-årig man som söker för magsmärta. Tilltagande buksmärta som flyttat sig åt höger i övre delen av buken. Haft besvären i ett par dagar. Tidigare sjukdomar: Inga. Mediciner: Inga. | 28-year-old man presenting with stomach ache. Increasing abdominal pain that has moved to the upper right part of the abdomen. Has had symptoms for a couple of days. Previous illnesses: None. Medication: None. | Immediate |
| 25 | 21-årig man som söker för ökad törst. Ofrivillig viktnedgång, ökade urinmängder. Tidigare sjukdomar: Inga. Mediciner: Inga. | 21-year-old man presenting with increased thirst. Unintentional weight loss, increased urine output. Previous illnesses: None. Medication: None. | Promptly |
| 26 | 12-årig flicka som söker för viktnedgång. Har ökad törst och miktionsfrekvens. Huvudvärk och buksmärta. Tidigare sjukdomar: Inga. Mediciner: Inga. | 12-year-old girl presenting with weight loss. Has increased thirst and frequent urination. Headache and abdominal pain. Previous illnesses: None. Medication: None. | Promptly |
| 27 | 79-årig man som söker för andfåddhet. Har frossa, diarre. Pat uppfattar att hans hud är blåaktig. Pat själv misstänker matförgiftning. Tidigare sjukdomar: Inga. Mediciner: Inga. | 79-year-old man presenting with shortness of breath. Has chills and diarrhea. The patient perceives his skin as blue-tinged, and suspects food poisoning. Previous illnesses: None. Medication: None. | Immediate |
| 28 | 44-årig kvinna som söker för smärta. Värk höger sida av rumpan samt utstrålning ner i ben. Smärtan är intensiv och olidlig. Kan ej sitta, gå eller sova. Smärtlindring hjälper ej. Fått barn. Inga komplikationer efter förlossning. Haft besvären i cirka en vecka. Tidigare sjukdomar: Oklart . Mediciner: Inga. | 44-year-old woman presenting with pain. Has a pain in the right buttock, radiating down the leg. The pain is intense and excruciating. Can't sit, walk or sleep. Pain killers do not help. Recently had a baby. No complications after delivery. Has had symptoms for about a week. Previous illnesses: Unknown. Medication: None. | Immediate |
| 29 | 21-årig man som söker för feber, kräkningar och allmänpåverkan (blek och kallsvettig, eller jobbigt med andningen). Tidigare sjukdomar: Inga. Mediciner: Inga. | 21-year-old man presenting with fever, vomiting and constitutional symptoms (pale and cold sweats, or difficulty breathing). Previous illnesses: None. Medication: None. | Immediate |
| 30 | 72-årig man som söker för bröstsmärta. Huggande och obehagskänsla i bröstet som kommer och går. Främst vid rörelse men även vid gång uppför trappa. Släpper i vila. Tidigare sjukdomar: Inga. Mediciner: Inga. | 72-year-old man presenting with chest pain. Stabbing and discomfort in the chest that comes and goes. Mainly when moving but also when walking up stairs. Subsides when he rests. Previous illnesses: None. Medication: None. | Immediate |
| 31 | 47-årig fullgången kvinna som söker för graviditetsbesvär. Har kräkningar/diarre samt förlossningsvärkar. Tidigare sjukdomar: Inga. Mediciner: Inga. | 47-year-old woman presenting with for pregnancy-related complaints. Is full-term. Has vomiting/diarrhea and pain, as well as contractions. Previous illnesses: None. Medication: None. | Immediate |
| 32 | 90-årig man som söker för synpåverkan. Akut insättande synfältsbortfall av höger synfält på båda ögonen. Plötslig symptomdebut. Tidigare sjukdomar: Oklart. Mediciner: Inga. | 90-year-old man presenting with visual impairment. Acute onset loss of right sided visual field in both eyes. Sudden onset of symptoms. Previous illnesses: Unknown. Medication: None. | Immediate |
| 33 | 46-årig kvinna som söker för yrsel. Stress, migrän, svartnar för ögon. Även viktnedgång och äter för lite. Haft besvären i sex veckor. Tidigare sjukdomar: Inga. Mediciner: Inga. | 46-year-old woman presenting with dizziness. Has experienced stress, migraine and visual black out. Has recently lost weight and is not eating properly. Has had symptoms for six weeks. Previous illnesses: None. Medication: None. | Planned |
| 34 | 50-årig man som söker för nytillkommen synpåverkan. Streck, svarta prickar i synfältet, blixtar med grå skugga i synfältet. Haft besvären i några dagar. Tidigare sjukdomar: Inga. Mediciner: Inga. | 50-year-old man presenting with recent visual impairment. Has lines and black dots in the visual field, flashes with a gray curtain. Has had symptoms for a few days. Previous illnesses: None. Medication: None. | Promptly |
| 35 | 32-årig gravid kvinna som söker för graviditetsbesvär. Illamående, huvudvärk, tryckkänsla i magen, vattenavgång, slem, lite blod, sammandragningar. Tidigare sjukdomar: Inga. Mediciner: Inga. | 32-year-old pregnant woman presenting with problems related to the pregnancy. Nausea, headache, feeling of pressure in the stomach. Waters have broken, with mucus and some blood. Has contractions. Previous illnesses: None. Medication: None. | Immediate |
| 36 | 65-årig kvinna som söker för huvudvärk. Svart fläck framför ögat. Tidigare sjukdomar: Inga. Mediciner: Inga. | 65-year-old woman presenting with headache. Has a black spot in the visual field. Previous illnesses: None. Medication: None. | Immediate |
| 37 | 39-årig kvinna som söker för smärta. Nack- och axelvärk med utstrålning i armarna. Tidigare sjukdomar: Diabetes. Mediciner: Insulin. | 39-year-old woman presenting with pain. Neck and shoulder pain with radiation in the arms. Previous illnesses: Diabetic. Medication: Insulin. | Immediate |
| 38 | 27-årig kvinna som söker för blödning under graviditet. Är tidigt i graviditeten. Har lågt sittande buksmärta höger sida. Tidigare sjukdomar: Inga. Mediciner: Inga. | 27-year-old woman presenting with vaginal bleeding during pregnancy. First trimester pregnancy. Low right-sided abdominal pain. Previous illnesses: None. Medication: None. | Immediate |
| 39 | 55-årig kvinna som söker för fumlighet. Besvär vänster hand. Huvudvärk. Haft besvären i några timmar. Tidigare sjukdomar: Förmaksflimmer. Mediciner: Blodförtunnande. | 55-year-old woman presenting with lack of coordination. Has symptoms in her left hand. Has a headache. Has had symptoms for a few hours. Previous illnesses: Atrial fibrillation. Medication: Anticoagulants. | Immediate |
| 40 | 25-årig gravid kvinna som söker för graviditetsbesvär. Har ej känt fosterrörelser under dagen. Tidigare sjukdomar: Inga. Mediciner: Inga. | 25-year-old pregnant woman presenting with pregnancy complaints. Hasn't felt fetal movements during the day. Previous illnesses: None. Medication: None. | Immediate |
| 41 | 56-årig man som söker för svaghet i arm. Har nedsatt kraft i höger arm. Känner sig snurrig. Haft besvären i några timmar. Tidigare sjukdomar: Inga. Mediciner: Inga. | 56-year-old man presenting with arm weakness. Has reduced strength in the right arm. Feeling dizzy. Has had symptoms for a few hours. Previous illnesses: None. Medication: None. | Immediate |
| 42 | 78-årig man som söker för bröstsmärta. Diffus bröstsmärta med utstrålning mot halsen. Tidigare sjukdomar: Inga. Mediciner: Inga. | 78-year-old man presenting with chest pain. Diffuse chest pain with radiation to the neck. Previous illnesses: None. Medication: None. | Immediate |
| 43 | 16-årig pojke som söker för ryggskada. Trauma - Hopp med cykel i crossbana - föll och landade på ryggen. Svår smärta i bröstrygg som strålade fram i bröstet samt stel i ryggen. Tidigare sjukdomar: Inga. Mediciner: Inga. | 16-year-old boy presenting with back trauma. Cross country bike jump - fell and landed on back. Severe pain in the upper back that radiates to the chest. Back stiffness. Previous illnesses: None. Medication: None. | Immediate |
| 44 | 40-årig man som söker för knöl på halsen. Tidigare sjukdomar: Inga. Mediciner: Inga. | 40-year-old man presenting with a lump on the neck. Previous illnesses: None. Medication: None. | Planned |
| 45 | 85-årig man som söker för svaghet i arm. Neurologiska bortfall. Small till i huvudet sedan blev höger arm och ben svaga. Tidigare sjukdomar: Inga. Mediciner: Inga. | 85-year-old man presenting with arm weakness. Neurological deficits. Sudden onset of headache, after which the right arm and leg became weak. Previous illnesses: None. Medication: None. | Immediate |
| 46 | 47-årig man som söker för pågående bröstsmärta som som debuterade föregående kväll. Tidigare sjukdomar: Inga. Mediciner: Inga. | 47-year-old man presenting with ongoing chest pain that began the previous evening. Previous illnesses: None. Medication: None. | Immediate |
| 47 | 78-årig man som söker för tryck över bröstet. Utstrålning vä armhåla, illamående, frusen. Haft besvären i tre dagar. Tidigare sjukdomar: Inga. Mediciner: Inga. | 78-year-old man presenting with tightness in chest. Radiates to right armpit. Is nauseous and feels cold. Has had symptoms for three days. Previous illnesses: None. Medication: None. | Immediate |
| 48 | 78-årig kvinna som söker för synpåverkan. Op gråstarr hö öga - efter två veckor debut av akuta besvär av värk, kraftig synnedsättning, rodnad och svullnad. Tidigare sjukdomar: Inga. Mediciner: Inga. | 78-year-old woman presenting with visual impairment. Had cataract surgery right eye. Two weeks after surgery acute onset of pain, severe visual impairment, redness and swelling. Previous illnesses: None. Doctor: None. | Immediate |
| 49 | 30-årig man som söker för känselbortfall. Nyligen vårdats för intracerebral blödning - nu nedsatt känsel i ena armen, domningar och pirrningar. Tidigare sjukdomar: Inga. Mediciner: Inga. | 30-year-old man presenting with sensory impairment. Recently had a intracerebral hemorrhage. Now sensory impairment in one arm, numbness and tingling. Previous illnesses: None. Medication: None. | Immediate |
| 50 | 75-årig man som söker för bensvullnad. Ömhet och svullnad i höger knä och underben. Haft besvären i ett par dagar. Tidigare sjukdomar: Lungemboli. Mediciner: Avslutat beh med blodförtunnande för två veckor sedan. | 75-year-old man presenting with swollen leg. Is sore, and has a swelling in the right knee and lower leg. Has had symptoms for a couple of days. Previous illnesses: Pulmonary embolism. Medication: Completed treatment with anticoagulants two weeks ago. | Promptly |
| 51 | 68-årig man som söker för bröstsmärta. Nytillkommen bröstsmärta vid ansträngning samt andfåddhet sedan ett par månader. Tidigare sjukdomar: Oklart. Mediciner: Oklart. | 68-year-old man presenting with chest pain. New chest pain on exertion and shortness of breath since a couple of months. Previous illnesses: Unknown. Medication: Unclear. | Immediate |
| 52 | 71-årig kvinna som söker för känselbortfall. Hela vänster sida ansikte, öra, arm, mage och ben domnat. Lite snurrig. Haft besvären ett par timmar. Tidigare sjukdomar: Diabetes, glutenallergi, astma, struma. Mediciner: Inga. | 71-year-old woman presenting with sensory loss. Entirely numb on her left side: face, ear, arm, stomach and leg. A little dizzy. Has had symptoms for a couple of hours. Previous illnesses: Diabetes, gluten allergy, asthma, goiter. Medication: None. | Immediate |
| 53 | 28-årig kvinna som söker för synpåverkan. Successivt mörknande i höger öga. Mörk punkt ses i yttre synfältet. Punkt blev större och större och sen försvann synen helt. Tidigare sjukdomar: Inga. Mediciner: Inga. | 28-year-old woman presenting with visual impairment. Gradual darkening in right eye, dark spot at the outer visual field. The spot got bigger and bigger and then her vision disappeared completely. Previous illnesses: None. Medication: None. | Immediate |
| 54 | 81-årig kvinna som söker för bröstsmärta. Tryck över bröstet, kallsvettig samt ökad andfåddhet vid ansträngning. Haft besvären i ett dygn. Tidigare sjukdomar: Inga. Mediciner: Inga. | 81-year-old woman presenting with chest pain. Pressure over her chest, cold sweats and increased shortness of breath on exertion. Experienced symptoms for a day. Previous illnesses: None. Medication: None. | Immediate |
| 55 | 24-årig man som söker för kräkningar. Kräkningar, "jätteskruttig", ser ut att ha gått ner 3 kg, kissar dåligt, senaste kräkningen luktar avföring. Haft besvären i 13 timmar. Tidigare sjukdomar: Som nyfödd op pga gastroschisis. Mediciner: Inga. | 24-year-old man presenting with vomiting. "In bad shape", seems to have lost 3 kg, urinating less than normal, last vomit smells like faeces. Has had symptoms for 13 hours. Previous illnesses: Operation as infant due to gastroschisis. Medication: None. | Immediate |
| 56 | 79-årig kvinna som söker för synpåverkan. Plötsligt svart för höger öga, synbortfall. Stora pupiller. Ljuset återkom med hälften ljust och hälften mörkt. Nu har besvär gått tillbaka. Besvären debuterade plötsligt. Tidigare sjukdomar: Frisk. Mediciner: Inga. | 79-year-old woman presenting with visual impairment. Suffered sudden loss of vision in the right eye. Large pupils. The vision returned with half of the field being bright and the other half darker. Symptoms have now entirely subsided. Sudden onset of symptoms. Previous illnesses: Healthy. Medication: None. | Immediate |
| 57 | 63-årig man som söker för tankar på att ta livet av sig. Ska ta livet av sig genom att hoppa från balkongen. Druckit mycket alkohol. Tidigare sjukdomar: Alkoholmissbruk. Mediciner: Inga. | 63-year-old man presenting with suicidal thoughts. Planning to kill himself by jumping from a balcony. Has drunk large amounts of alcohol. Previous illnesses: Alcohol abuse. Medication: None. | Immediate |
| 58 | 35-årig gravid kvinna som söker för graviditetsbesvär. Fullgången graviditet. Nu blödning, värkar och sammandragningar. Tidigare sjukdomar: Infertilitetsproblem, depression. Mediciner: Inga. | 35-year-old pregnant woman with a full-term pregnancy presenting with bleeding, pains and contractions. Previous illnesses: Infertility problems, depression. Medication: None. | Immediate |
| 59 | 75-årig kvinna som söker för svaghet. Ramlat ur sängen och fått hjälp av trygghetslarmets personal tidigare samma morgon. Nu skakar i kroppen, kan ej stödja på benen. Dagen innan haft synfältsbortfall. Nya besvär sedan i morse. Tidigare sjukdomar: Diabetes. Mediciner: Inga. | 75-year-old woman presenting with weakness. Fell out of bed and received help from the home care staff earlier that morning. Now experiencing a general shakiness and inability to support herself on her legs. Sufferred vision loss the day before. New symptoms since this morning. Previous illnesses: Diabetes. Medication: None. | Immediate |
| 60 | 48-årig man som söker för besvär efter operation. Op ljumskbråck med nät höger sida. Dagen efter frossa och stigande temp. Tidigare sjukdomar: Op för samma tidigare. Mediciner: Inga. | 48-year-old man presenting with discomfort after surgery. Operated inguinal hernia with mesh on the right side. Following day chills and rising temp. Previous illnesses: Previously undergone same operation. Medication: None. | Immediate |
| 61 | 48-årig man som söker för känselbortfall. Stickningar och domningar i vänster arm och stickningar vä ben. Dålig styrsel i vä sida som märks vid gång. Haft besvären i 15 minuter. Tidigare sjukdomar: Inga. Mediciner: Inga. | 48-year-old man presenting with sensory loss. Tingling and numbness in the left arm and tingling in the left leg. Poor control of left side which is noticeable when walking. Has had symptoms for 15 minutes. Previous illnesses: None. Medication: None. | Immediate |
| 62 | 32-årig kvinna som söker för graviditetsbesvär. Förstföderska med sammandragningar som ej går över vid vila. Tidigare sjukdomar: Inga. Mediciner: Inga. | 32-year-old woman presenting with pregnancy-related complaints. First-time pregnancy, has contractions that do not go stop when resting. Previous illnesses: None. Medication: None. | Immediate |
| 63 | 11-årig pojke som söker för ökad törst. Dricker och kissar ovanligt mycket. Allmäntillstånd ua. Haft besvären i ett par veckor. Tidigare sjukdomar: Inga. Mediciner: Inga. | 11-year-old boy presenting with increased thirst. Is drinking and urinating more than usual. No constitutional symptoms. Had the symptoms for a couple of weeks. Previous illnesses: None. Medication: None. | Promptly |
| 64 | 3-årig pojke som söker för ökad törst. Har druckit och kissat mkt. Opåverkad. Haft besvären i en dag . Tidigare sjukdomar: Oklart . Mediciner: Inga. | 3-year-old boy presenting with increased thirst. Has been drinking and urinating a lot. No other symptoms. Has had the symptoms for a day. Previous illnesses: Unknown. Medications: None. | Promptly |
